# Adoption of Alcohol Health Warning Labels (AHWLs) across South East Asia: current status and recommendations for future

**DOI:** 10.1016/j.lansea.2025.100557

**Published:** 2025-03-10

**Authors:** Swarndeep Singh, Yatan Pal Singh Balhara

**Affiliations:** a Department of Psychiatry, VMMCH and Safdarjung Hospital, New Delhi, India; b National Drug Dependence Treatment Center and Department of Psychiatry, All India Institute of Medical Sciences (AIIMS), New Delhi, India

## Introduction

Harm due to alcohol use is a great public health concern for the South East Asia region (SEAR) with the annual per capita consumption expected to grow even further.

Health-related information labelling on alcohol products has been recommended as a public health strategy to address the problems associated with the use of alcohol.^1^ Ireland is on its way to become the first country in the world to require comprehensive health warnings on alcohol products by the year 2026.^2^ Recommendations have been made to include cancer warning labels on alcoholic beverages.^3^^,^^4^ However, less than one-third of all the countries surveyed globally have regulations mandating Alcohol Health Warning Labels (AHWLs) on alcohol containers.^1^ Moreover, the content as well as its presentation are critical determinants of the potential impact of these health-related information labelling on the subsequent consumption of alcohol products.^5^

The data captured by the Global Health Observatory data repository of the WHO offers information on the status of AHWLs across the countries in the SEAR.^6^ In addition, the reports published by the WHO on Alcohol Policy with mention of AHWLs also offers information regarding the status of AHWLs across these countries.^7^^,^^8^ Also, an attempt was made to extract information from government websites and other publicly available online sources of information regarding the current status of AHWLs across these countries. The status of the indicators for tracking the implementation status of AHWLs has been presented in Table 1.

**Table 1:** Status of adoption of the Alcohol Health Warning Labels (AHWLs) across the South East Asian countries.^a^

	Bangladesh	Bhutan	India	Indonesia	Myanmar	Sri Lanka	Thailand	Timor-Leste
Health warning labels on alcohol containers	No data	No	Yes	No/Yes^b^	No	No	Yes	No
Health warning labels on alcohol advertising	No data	No	Yes	No/Yes^b^	No	No	Yes	No
Consumer information about calories, additives, etc on containers	No	Yes- wine, beer, spirits	No	No	No	No	No	No
Number of standard alcoholic drinks displayed on containers	No	No	No	No	No	No/Yes^b^	No/Yes^b^	No
Alcohol content displayed on containers	Yes- beer, wine, spirits	Yes- beer, wine, spirits	Yes- beer, wine, spirits	Yes- beer, wine, spirits	No	Yes- beer, wine, spirits	Yes- beer, wine, spirits	No
Health warning labels on pregnancy	No	Not applicable	No	No	Not applicable	Not applicable	No	Not applicable
Health warning labels on under-age drinking	No	Not applicable	No	No	Not applicable	Not applicable	Yes	Not applicable
Health warning labels on drink-driving	No	Not applicable	No	No	Not applicable	Not applicable	Yes	Not applicable
Legal requirement for size of health warning labels	No data	No data	No	No data	Not applicable	Not applicable	Yes	Not applicable

a Based on Global Health Observatory data repository of the WHO and reports published by the WHO on Alcohol Policy with mention of AHWLs. No data were available for DPR Korea, Maldives and Nepal.

b Listed as present in the Report on the Alcohol Policy in the WHO South-East Asia Region by WHO.

We can draw some important inferences from available data on the indicators of AHWLs implementation in the SEAR.

1. With the exception of Thailand, the adoption of AHWLs in the South East Asian countries remains limited.
2. Some of the well-established adverse health consequences associated with alcohol use such as harmful health consequences of alcohol consumption related to fatal cancers, liver damage and other non-communicable diseases and road traffic accidents have not yet been included in the AHWLs.
3. AHWLs that are of relevance to the populations with specific needs have not been adopted in most of the countries. These include health warning labels related to pregnancy and health warning labels on under-age drinking.
4. Consumer information about calories, additives (including allergens), etc. on containers is missing in most of the South East Asian countries.
5. While displaying the alcohol content on the container has been adopted in most of the countries, the practice of mentioning the number of standard alcoholic drinks on the container is yet to be adopted in many countries.
6. The legal requirement for size of health warning labels are yet to be adopted in most of the countries. Various design factors such as size, placement, colour, pictorials, length, signal words like “warning” or “health warning” and physical interactivity has been shown to influence the consumers' attention.^9^

It has been recommended that use of AHWLs along with other alcohol control measures to increase awareness about various harms associated with alcohol use could help people make more informed decisions.^1^ We propose that mandatory AHWLs should be implemented along with other effective alcohol control strategies in all countries. We have tabulated the AHWL messages (see Table 2) that have been found to be effective in a recent systematic review in at least one real world study.^10^ The evidence supports use of AHWLs along with other effective alcohol control policy measures to mitigate alcohol use related harms.^6^ Further, a QR code could also be provided on each container with health warning message on the AHWL that could allow the person to access more elaborate infographic(s) or short audio-video messages on alcohol related harms in various local languages. A recent study from Spain tested the effects of displaying QR codes with alcohol related health information on prominently displayed banners at the alcohol section of a supermarket, and reported a low usage rate of 2.6 per 1000 among individuals who purchased alcohol.^11^

**Table 2:** Alcohol Health Warning Label (AHWL) messages that have been found to be effective as per the available evidence base.

Message theme/content	Type of presentation
Standard government label, alcohol-related self-affirmation	Text warning
Cardiovascular, liver damage, addiction, capacity, tension, Alcohol-related Harms, stress, loneliness	Text warning
Birth defects, driving impairment, operating machinery, Alcohol-related diseases	Text warning
Cancer, national drinking guidelines, standard drinking information	Text warning
Birth defects, driving impairment, operating machinery, Alcohol-related diseases, cancer, poison, toxic	Text warning
Birth defects, driving impairment, operating machinery, smoking, carbon monoxide, coffee, tea	Text warning
Pregnancy, driving impairment and operation of machinery, health problems	Text warning
Cancer, national drinking guidelines, standard drinking information	Text warning
National low risk drinking guidelines, standard drinking information	Text warning
Cancer, national low risk drinking guidelines, standard drinking information	Text warning
Birth defects	Pictorial warning
Alcohol-related diseases	Text warning, Pictorial warning

Alcohol labelling practices does not follow any standard guidelines across different countries. Further, even in countries where AHWLs are present on containers, they are not in line with the best-practice recommendations. The available evidence for effectiveness of AHWLs and factors affecting its success are not well-established due to both limited number of studies and often contradictory or mixed findings reported about the effect of AHWLs on reducing alcohol use and related harms across different studies. The inconsistencies or deficiencies in the implementation of AHWLs in terms of the design, formatting, and content of the health warning message displayed on alcohol containers across different studies might also be an important contributory factor behind these mixed findings.

Major barriers in effective implementation of alcohol control policies include exploitation of legal loopholes by alcohol-industry; low priority of policy implementation among responsible agencies; and insufficient experience or resources for ensuring their effective implementation. Available literature suggests that World Trade Organization (WTO) discussions at Technical Barriers to Trade (TBT) committee meetings often advanced alcohol-industry supported arguments against adoption of potentially effective alcohol control policies such as implementation of best-practice alcohol labelling policies making it mandatory for the manufacturers to display AHWLs as per the WHO's recommendation.^12^ For example, Thai government's attempt to make graphical AHWLs covering at least 30% of alcohol container at front side similar to that found on tobacco packages was blocked after WTO intervention and international alcohol-industry. Arguments put forward by them were mainly centred around legal loopholes in trade agreements and lack of adequate empirical evidence supporting its effectiveness or appropriateness for all types of alcoholic beverages or consumption patterns (e.g., moderate consumption of red wine). Thus, need for having increased transparency about vested interests of WTO participants at meetings discussing alcohol industry regulations is strongly advocated.

In conclusion, the adoption of the AHWLs across the South East Asian countries is variable. There is a need to invest in identification of evidence based AHWLs, their effective implementation and sharing of the data on its implementation.

## Contributors

SS and YPSB conceptualised the article, collected the data, prepared the first draft, revised the draft, approved the final draft.

## Declaration of interests

The authors have no conflict of interest to declare.
